# Determination of Aflatoxin M_1_ , Organochlorine Pesticides, and Heavy Metals in Raw Milk of Iran

**DOI:** 10.1002/fsn3.71135

**Published:** 2025-10-26

**Authors:** Farnoosh Ansari, Elaheh Askari, Hamdollah Naderi Boroujeni, Maliheh Jahanara, Bita Forootani, Elham Khalili Sadrabad

**Affiliations:** ^1^ Research Center for Food Hygiene and Safety, Department of Food Hygiene and Safety, School of Public Health Shahid Sadoughi University of Medical Sciences Yazd Iran; ^2^ Pegah Infant Formula Company Shahrekord Iran; ^3^ Nutritional Health Research Center, School of Health and Nutrition Lorestan University of Medical Sciences Khorramabad Iran; ^4^ Shiraz University Shiraz Iran; ^5^ Department of Nutrition, School of Public Health Shahid Sadoughi University of Medical Sciences Yazd Iran; ^6^ Department of Food Hygiene and Safety, School of Public Health Shahid Sadoughi University of Medical Sciences Yazd Iran

**Keywords:** aflatoxin M_1_, heavy metals, organochlorine pesticides, raw milk

## Abstract

The current study aimed to investigate aflatoxin M_1_ (AFM_1_), organochlorine pesticides (OCPs), and heavy metals (Cd, As, Pb, Hg) in raw milk (50 samples) from Milk Company, Shahrekord Province, Iran. Therefore, AFM_1_, OCPs, and heavy metals including Pb, Cd, As, and Hg were analyzed by High‐Performance Liquid Chromatography (HPLC), Gas Chromatography–Mass Spectrometry (GC–MS), and Graphite Atomic Absorption Spectrophotometry, respectively. According to the results, AFM_1_ in all milk samples was lower than the maximum limits set by Codex Alimentarius Commission (0.05 μg/kg), Iranian Standard (100 ng/L), and European Union standard (0.05 μg/kg). The AFM_1_ in milk samples varied from 1 < to 92 ng/L and 1 < to 27 ng/L in spring and summer seasons, respectively. The concentrations of OCPs in all milk samples were lower than standard limits. According to the results, α‐HCH, β‐HCH, γ‐HCH varied from > 0.01–0.03 and > 0.01, > 0.02–0.04 and > 0.01–0.011, and > 0.01–0.02 and > 0.01–0.013 mg/kg in spring and summer respectively. DDE, DDD, and DDT varied from lower than 0.005 mg/kg (>LOD) to 0.013 mg/kg (*p* > 0.05). The mean concentration of Pb, Cd, As, and Hg in spring and summer was 0.007 and 0.014 mg/L, 0.011 and 0.005 mg/L, 0.008 and 0.011 mg/L, 0.002 and 0.002 mg/L, respectively. The study confirmed that AFM_1_, OCPs, and heavy metal residues in milk samples were within safe limits set by regulatory authorities, with slight seasonal variations. Although contamination levels were low, ongoing surveillance is essential to ensure dairy safety and minimize potential health risks.

## Graphical abstract

The consumption of milk by different age groups makes its contamination as a crucial issue for public health. Therefore, the consumption of milk is considered in terms of AFM1, OCs, and heavy metals. Although monitoring of the milk safety is necessary.

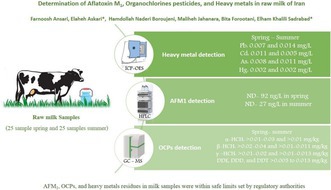

**Abbreviations**
AFB_1_
: aflatoxin B_1_
AFM_1_
: aflatoxin M_1_
DDD: dichlorodiphenyldichloroethane
DDE: dichlorodiphenylchloroethylene
DDT: dichlorodiphenyltrichloroethane
GC–MS: gas chromatography–mass spectrometry
HPLC: high‐performance liquid chromatography
ICP‐OES: inductively coupled plasma‐optical emission spectrophotometry
LOD: limit of detection
LOQ: limit of quantification
OCPs: organochlorine pesticides
α‐HCH: α‐ hexachlorocyclohexane
β‐HCH: β‐ hexachlorocyclohexane
γ‐HCH: γ‐ hexachlorocyclohexane

## 1 Introduction

Milk is a nutritionally essential food, particularly for infants and young children, owing to its high content of proteins, lipids, lactose, calcium, vitamins, and antioxidants (Patyal et al. 2020; Kaur et al. 2021). Due to the widespread consumption of milk by various age groups, especially sensitive populations such as infants and the elderly, its contamination poses significant public health risks. Among potential contaminants, milk can be contaminated by biological and chemical hazards, including aflatoxins (AFs), pesticide residues, antibiotic residues, and heavy metals.

Aflatoxins, a difuranocoumarin derivative mycotoxin, are secondary metabolites produced primarily by *Aspergillus parasiticus* and *Aspergillus flavus* (Patyal et al. 2020) which contaminated 25% of global foodstuffs annually (Hajmohammadi et al. 2020). By contamination of livestock feed with aflatoxin B_1_ (AFB_1_), it is absorbed in the gastrointestinal tract, metabolized in the liver into aflatoxin M_1_ (AFM_1_), and subsequently excreted into milk (Daou et al. 2020; Patyal et al. 2020). The studies indicated that 0.3% to 6.2% of ingested AFB_1_ by contaminated feed is bio‐transformed into AFM_1_ which could be detectable in milk after 12–24 h ingestion (Patyal et al. 2020). Unfortunately, the high heat resistance of AFM_1_ due to the capability of AFM_1_ to bind to milk casein (Hajmohammadi et al. 2020) makes it more dangerous for human health. Due to its proven carcinogenic, hepatotoxic, and immunosuppressive properties, mutagenic, and teratogenic effects (Kamkar, Fallah, and Mozaffari Nejad 2014), AFM_1_ poses serious public health risks, particularly to elderly people, infants, and children who are major consumers of milk (Tarannum et al. 2020). According to the International Agency for Research on Cancer (IARC), AFM_1_ has been introduced as a potential human carcinogen Group 1 (Mousavi Khaneghah et al. 2023).

The presence of pesticide residues, particularly organochlorine pesticides (OCPs), in milk is due to the consumption of contaminated feed and fodder by animals (Nag 2010). Organochlorine pesticides with the capability to accumulate in fatty tissue could enter the different organs, including the mammary gland (Nag 2010). Organochlorine pesticides, including DDT (Dichlorodiphenyltrichloroethane) and HCHs (hexachlorocyclohexane), could disrupt the endocrine system, cause carcinogenic effects, and damage the nervous and immune systems (Manav et al. 2019).

Milk contains several elements such as copper, zinc, and iron, which are essential for human metabolism. Otherwise, the presence of toxic heavy metals, including cadmium, arsenic, lead, and mercury, could lead to metabolic disorders. Due to the effects of heavy metals on milk composition, as well as human health problems, monitoring of milk for contamination could be an environmental indicator for the detection of probable health hazards (Monteverde et al. 2022).

In recent years, chemical and biological contamination of the environment (air, soil, and water) and feed resulted in their entrance into the food chain. Due to the consumption of milk by a wide range of age groups, the investigation of the safety and probable presence of chemical contaminants is a crucial issue. Therefore, the purpose of the current study was to determine the AFM_1_, organochlorine pesticides, and heavy metals (Pb, Cd, As, and Hg) in the raw milk of Iran.

## 2 Materials and Methods

### 2.1 Milk Sample Preparation

This cross‐sectional study involved the random collection of 50 raw milk samples from Milk Company, Shahrekord province, Iran. Samples were collected between April and September 2023, of which 25 samples were randomly gathered from each season (spring and summer). All collected samples were transported to the laboratory and stored at 4°C until further analysis (Nejad et al. 2019).

### 2.2 Aflatoxin M_1_

#### 2.2.1 Sample Extraction

The milk samples were extracted according to the method explained by Alahlah et al. (2020) with some modifications. To eliminate the fat layer, 60 mL of milk samples were centrifuged at 3000 × ***g*** for 10 min. Then, the defatted samples were cleaned by an immuno‐affinity column and washed with PBS (Phosphate Buffer Saline, 10 mL). To elute AFM_1_, the acetonitrile‐methanol solution (60:40 v/v) was used. To evaporate the solution, samples were kept under a nitrogen stream in darkness (50°C), and the residues were used for HPLC analysis.

#### 2.2.2 Sample Analysis

**FIGURE 1:**
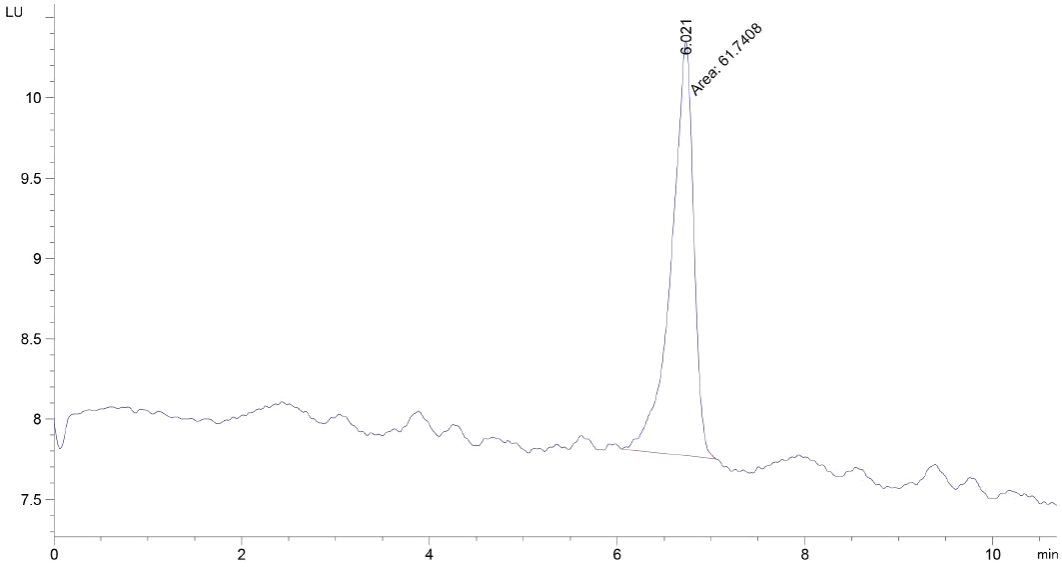
HPLC peaks of aflatoxin M_1_.

The aflatoxin M_1_ was measured according to AOAC 2000.08–2004 by HPLC (Figure 1) (AOAC 2005). The 50 μL sample was injected to HPLC (Agilent coupled with fluorescence detector, column C_18_, excitation at 360, and emission at 440 nm) with a flow rate of 0.8 mL/min. The acetonitrile‐water in a ratio of 25%–75% V/V was used as a mobile phase (Hooshfar et al. 2020).

### 2.3 Method Validation

The calibration curve was prepared by different standard solutions of AFM_1_ (0.05, 0.1, 0.2, 0.5, and 1.0 μg/L) and recorded peak areas by injection to HPLC. The standard solutions were injected to HPLC several times to calculate the standard deviation (*σ*), and LOD and LOQ were evaluated by the following formula:

$$\mathrm{LOD}=3.3\times \left(\sigma /S\right)$$

$$\mathrm{LOQ}=10\times \left(\sigma /S\right)$$

where *S* is the slope of the calibration curve.

Also, a spiked recovery test (0.25, 0.5, 0.75, 1, 1.25, and 1.5 μg/L AFM_1_, and blank milk sample) was used to validate method accuracy (acceptable recovery: 70%–120%) (Mashak et al. 2016). In the current study, the recovery test was 112.14% for AFM_1_, which was in the acceptable range. Also, LOQ and LOD were 0.05 ng/g and 1 ng/L, respectively.

### 2.4 Organochlorine Pesticides

#### 2.4.1 Sample Extraction

Approximately 10 mL of homogenized milk samples were added to a tube containing 20 mL n‐hexane: acetone (ratio of 1:1) and 1 g sodium chloride. The mixture was shaken for 5 min, incubated in an ultrasonic bath for 10 min, and centrifuged at 1500 × ***g*** for 5 min. The supernatant was separated and then 100 mL of anhydrous sodium sulfate was added and evaporated in a rotary evaporator. To remove the fat, extracts were dissolved in *n*‐hexane (15 mL) and sulfuric acid (2 mL). Then, the sulfuric acid was removed by washing with 10 mL sodium sulfate liquid solution. The *n*‐hexane (1 mL) was added to remove the organic layer and filtered through a 0.22 μm membrane filter before GC–MS analysis (Hasan et al. 2022).

#### 2.4.2 Sample Analysis

2 μL of extracted samples were injected to a GC–MS system (Agilent 7890A with 5975C MSD) in which helium gas with a flow rate of 1.2 mL was used as the carrier gas. The temperature of the system ranged from 85°C to 290°C.

#### 2.4.3 Method Validation

The validation determined recovery and limit of detection (LOD) to ensure method accuracy and sensitivity. In the current study, the LODs were α‐HCH (α‐Hexachlorocyclohexane), β‐HCH (β‐Hexachlorocyclohexane), γ‐HCH (γ‐Hexachlorocyclohexane) = 0.01 mg/kg, p,p'‐DDE (p,p'‐Dichlorodiphenylchloroethylene), p,p'‐DDD (p,p'‐Dichlorodiphenyldichloroethane), p,p'‐DDT (p,p'‐Dichlorodiphenyltrichloroethane) = 0.005 mg/kg, Heptachlor = 0.001 mg/kg. The recovery (recovery of 70% to 120% and RSD ≤ 20%) was done by spiking blank milk containing five concentrations of pesticide standards (0.5 to 200 μg/kg) with three replicates for each level. The recovery for α‐HCH, β‐HCH, γ‐HCH, p,p'‐DDE, p,p'‐DDD, p,p'‐DDT, Heptachlor was 98.21%, 99.87%, 84.37%, 97.46%, 89.58%, 99.83%, and 85.37%, respectively.

#### 2.4.4 Sample Preparation

The milk sample for As, Cd, and Pb analysis (2 mL) was digested in the presence of 2 mL HNO_3_ (65%) and 1 mL H_2_O_2_ (30%). The digestion was heated in closed vessels at 90°C for 2 to 3 h. After filtration of the digested samples, they were diluted to 10 mL by distilled deionized water (Alipour et al. 2023). For the preparation of samples for Hg analysis, the milk samples (2 mL) were digested by 2 mL HNO_3_ (65%) and sulfuric acid in the presence of potassium permanganate (5%).

#### 2.4.5 Sample Analysis

Heavy metals analyses were done by Inductively Coupled Plasma‐Optical Emission Spectrophotometer (ICP‐OES, Spectro Genesis model). The corresponding wavelength for analysis of As, Cd, and Pb in milk samples was 189.042, 214.438, and 220.353 nm, respectively. The limit of detection (LOD) of samples was set at 0.698 ppb, 0.665 ppb, and 1.87 ppb for As, Cd, and Pb, respectively. Also, the cold vapor atomic absorption technique was used for Hg detection in milk samples (LOD = 0.541 ppb).

#### 2.4.6 Method Validation

The method accuracy of heavy metals analysis was done according to standard reference material (Kheirati Rounizi et al. 2021), in which the recovery values were 96.24%, 102.78%, 97.58%, and 105.92% for As, Cd, Pb, and Hg, respectively.

### 2.5 Statistical Analysis

The statistical analysis was done by SPSS 18.0 software. The data were analyzed by two‐way analysis of variance and reported as mean ± standard deviation. The Kruskal‐Wallis tests were applied for comparison of means.

## 3 Results and Discussion

### 3.1 Aflatoxin M_1_ in Milk

Owing to the cow lactation stage and milk production, about 0.3% to 6.2% of AFB_1_ is converted to AFM_1_ and secreted to milk (Hajmohammadi et al. 2020). According to the results of the current study (Table 1), the AFM_1_ in all milk samples was lower than the maximum limits set by the Institute of Standards and Industrial Research of Iran (ISIRI) (100 ng/L) and the Codex Alimentarius Commission (0.05 μg/kg) (Commission 2001). As results showed, the mean concentration of AFM_1_ in spring raw milk samples (20.06 ng/L) was significantly (*p* ≤ 0.05) higher than that in summer raw milk samples (11.76 ng/L). The mean concentration of AFM_1_ in milk samples gathered from Gorgan, Iran, in summer, spring, winter, and autumn was 43.38, 63.02, 78.83, and 36.82 ng/L, respectively (Rahimzadeh Barzoki et al. 2023). In the study by Bilandžić et al. (2017) the mean concentration of AFM_1_ in spring cow milk samples was 5.73 ng/kg, which is lower than the results of the current study. The AFM_1_ in spring milk samples of Tomašević et al. (2015) (375 ng/kg), Nemati et al. (2010) (52.9 ng/kg), and Xiong et al. (2013) (98 ng/kg) was higher than that in the spring samples of the present study. This difference could be related to the lower temperature of spring in comparison to summer and the low quality of feeding fodder for livestock in early spring (Xiong et al. 2013; Rahimzadeh Barzoki et al. 2023), including mixed complementary feed and lack of fresh green feeds (Bilandžić et al. 2022), which are adapted to the geographical and climate temperature of our studied region (cold mountainous area). Also, the temperature of 25°C to 37°C with 80% to 85% humidity is a favorable condition for aflatoxin‐producing fungi (Bilandžić et al. 2022).

**TABLE 1:** Concentration of aflatoxin M_1_ (ng/L) in raw milk samples.

Categories (2023 year)	Number of milk samples	Number of positive samples	Min – Max ng/L	Mean ± SD ng/L
Spring	25	21	> 1* to 92	20.06 ± 25.51^a^
Summer	25	18	> 1* to 27	11.76 ± 12.10^a^

*Note:* Different letters in two seasons show significant difference at *p* ≤ 0.05.

* LOD was 1 ng/L.

In the current study, AFM_1_ was found in 21 of the 25 spring samples and 18 of the 25 summer samples. The results of Mozaffari Nejad et al. (2019) confirmed the detection of AFM_1_ in 55 pasteurized milk samples and 21 UHT milk samples gathered from Hamedan province of Iran. The AFM_1_ in raw milk samples varied from 1 < −92 ng/L and 1 < −27 ng/L in spring and summer seasons, respectively. The mean value of AFM_1_ was reported as 14.76 ng/kg in the northern area of Morocco (Alahlah et al. 2020), 0.59 μg/L in Punjab, Pakistan (Akbar et al. 2020), 61 ng/L in Razavi Khorasan Province, Iran (Hajmohammadi et al. 2020), 0.314 μg/L in Ludhiana, Punjab (Kaur et al. 2021), 4.46 ng/L in Morocco (Mannani et al. 2021), 0.83 μg/kg in Bishoftu town of Ethiopia (Tadesse et al. 2020), 99.77 ng/kg (pasteurized milk) and 35.46 ng/kg (UHT milk) in Bangladesh (Tarannum et al. 2020), and 68.4 ng/L in Punjab, Pakistan (Waqas et al. 2021). Also, Mashak et al. (2016) reported AFM_1_ in a range of 0.015 to 0.14 μg/L in UHT flavored milk samples collected from Karaj, Iran, of which 33.3% of samples contained AFM_1_ higher than EU regulation and lower than ISIRI limits. In Kamkar, Yazdankhah, et al. (2014) study, AFM_1_ was detected in 69% (mean concentration of 55 ng/L) and 79% (mean concentration of 116 ng/L) of raw cow and buffalo milk samples gathered from Shush city of Iran, respectively, which were lower than ISIRI limits. According to the results of Patyal et al., the high mean (0.917 μg/L) concentration of AFM_1_ was reported (Patyal et al. 2020). In Jafari's et al. (2021) research, the mean AFM_1_ in industrial and traditional milk was evaluated as 54.33 ± 12.22 and 53 ± 11.49 ng/L, respectively. The variation in AFM_1_ across different studies could be related to the analysis method (HPLC or ELISA), sample size, season of sampling (summer or winter), and geographical conditions (Mannani et al. 2021). In addition, it seems that the occurrence of AFM_1_ contamination is more probable in drought years in comparison to non‐drought years (PEñA‐Rodas et al. 2018). Also, contamination of animal feed with aflatoxin B_1_ (AFB_1_), especially in stored fodders, could be related to the presence of AFM_1_ in milk samples. Moreover, humid weather, rainfall, annual temperature, use of industrial or self‐prepared foodstuff, improper storage conditions, or inadequate postharvest practices increase the probable growth of aflatoxigenic fungi and contamination of animal feed (Akbar et al. 2020, Kaur et al. 2021, PEñA‐Rodas et al. 2018). The quantity of feed consumed by cows, lactation phase, metabolic and health condition, cow breed, rumen microflora, mammary gland health (mastitis), hepatic biotransformation ability, and particle size of food could have an effect on AFM_1_ concentration (Zentai et al. 2023). In recent years, widespread application of adsorbents, including sodium bentonite, as well as polysaccharides and peptidoglycans of yeast cell walls, has led to a decrease in AFB bioavailability, which resulted in lower excretion of AFM_1_ into milk (Zentai et al. 2023). Therefore, proper farming management, such as adequate irrigation, good harvesting (maintain the integrity of crops or plants), post‐harvest practices (maintain adequate moisture of crops or plants), and clean feed containers, can reduce the contamination of feedstuff and growth of fungi (Waqas et al. 2021; Patyal et al. 2020). Also, the use of green fodder instead of soybean oil cake, sesame oil cake, wheat bran, or rice bran could reduce the risk of fungi contamination (Waqas et al. 2021).

### 3.2 Organochlorine Pesticides in Milk

Although the organochlorine pesticides (OCPs) have been banned since the 1980s, due to their high environmental stability and extensive usage in the past, the contamination of milk has been reported. Owing to the lipophilic nature of OCPs, their presence in milk fat is a controversial problem (Muhammad Arif et al. 2021). By the entrance of DDT into the mammalian body, the biotransformation of DDT into DDE and DDD will occur by dehydrochlorination and reductive dechlorination, respectively (Kuba et al. 2015).

The mean concentrations of γ–HCH and DDTs (Table 2) in spring milk samples were higher than in summer ones (*p* > 0.05). Although α‐HCH and β‐HCH of spring milk samples were significantly higher than in summer ones (*p* ≤ 0.05). According to Muhammad Arif et al. (2021) results, the lowest and highest OCPs concentrations in milk samples were shown in winter and the warmer month of the year, respectively. In the Ashoub and Azam study, the highest OCPs in milk samples were found in summer samples (Ashoub and Azam 2016), which are not in agreement with the results of the current study. The high amount of OCPs in the colder season and early spring could be due to the feeding of animals with oil seeds and their secretion into milk, which is compatible with the results of Muhammad Arif et al. (2021).

**TABLE 2:** Concentration of organochlorine pesticides (OCPs) in raw milk samples.

Categories (2023)	α‐HCH (mg/kg)	β‐HCH (mg/kg)	γ‐HCH (mg/kg)	p, p'‐DDE (mg/kg)	p, p'‐DDD (mg/kg)	p, p'‐DDT (mg/kg)	Heptachlor (mg/kg)
Spring	> 0.01–0.03	0.02–0.04	> 0.01–0.02	> 0.01–0.012	> 0.005–0.01	> 0.005–0.013	> 0.001–0.002
Summer	> 0.01	> 0.01–0.011	> 0.01–0.013	> 0.01–0.01	> 0.005–0.012	> 0.005–0.01	> 0.001–0.002
*p*	*p* ≤ 0.05		*p* > 0.05				

*Note:* *p* ≤ 0.05 shows significant difference among two seasons. LOD: α‐HCH, β‐HCH, γ‐HCH = 0.01 mg/kg, p,p'‐DDE, p,p'‐DDD, p,p'‐DDT = 0.005 mg/kg, Heptachlor = 0.001 mg/kg.

According to the European Union, the maximum residue of DDT and DDE in milk samples was determined to be 40 μg/kg. According to the Codex Alimentarius Commission, the maximum residue limit (MRL) of DDT, γ‐HCH, and heptachlor has been associated with 0.02, 0.01, and 0.006 mg/kg on a fat basis, respectively. As can be seen in Table 2, the concentrations of OCPs in all milk samples were lower than standard limits. According to the results, α‐HCH, β‐HCH, and γ‐HCH varied from > 0.01–0.03 and > 0.01, > 0.02–0.04 and > 0.01–0.011, and > 0.01–0.02 and > 0.01–0.013 mg/kg in spring and summer, respectively. The DDE, DDD, and DDT varied from ND or lower than 0.005 mg/kg to 0.013 mg/kg (*p* > 0.05). In the research on milk gathered from Romania, the average concentrations of α‐HCH, β‐HCH, γ‐HCH (lindane), heptachlor, and total DDTs were 1.1, 4.7, 1.9, 1.7, and 5.2 ng/g fat, respectively (Miclean et al. 2024). In the Derouiche et al. (2023) study, the averages of DDTs, HCB, and HCHs in milk samples were evaluated to be 17.6, 14.31, and 0.77 ng/g. The mean concentration of PCBs in Croatian milk samples ranged from 1.38 to 2.74 μg/kg (Đokić et al. 2024). The mean concentrations of α‐endosulfan, β‐endosulfan, endosulfan‐sulphate, DDE, γ‐HCH, and dieldrin in dairy farm milk samples (Lahore district, Pakistan) were found to be 26.94 ± 4.63, 59.88 ± 6.76, 32.07 ± 4.51, 4.64 ± 0.48, 1.20 ± 0.17, and 1.93 ± 0.18 μg/kg, respectively (Muhammad Arif et al. 2021). According to Sana et al., the OCPs varied between 3.93 and 27.63 ng/mL in buffalo milk, whereas cow's milk exhibited a higher range of 14.64–77.93 ng/mL. Also, the HCHs followed by DDTs were the predominant OCPs in cow and buffalo milk (Sana et al. 2021). In a study in Egypt, the total DDTs and HCH in milk samples were estimated to be 114 ± 12.4 and 140.6 ± 4.6 ng/g, respectively, which indicated the illegal use of these OCPs (Mohamed et al. 2024).

Due to the lipophilic character of OCPs, their bio‐accumulation in the animal body and their transfer to milk are crucial (Miclean et al. 2024). Municipal waste incinerators, contaminated feedstuffs, pollutants' bioavailability, atmospheric deposition in animal feed and water (Matei et al. 2023; Miclean et al. 2024), water adulteration in milk (Muhammad Arif et al. 2021), along with animal feed, and the grazing of animals could lead to the digestion of soil, which could be highly contaminated with OCPs (Miclean et al. 2024).

### 3.3 Heavy Metals in Milk

Determination of heavy metals in milk and dairy products can be a reflection of environmental pollution (Monteverde et al. 2022). The presence of heavy metals in milk samples could be due to industrial contamination of fodders and air contamination, which could transfer metals to the animal's body. Moreover, heavy metals contamination in animal feed can transfer these metals into the animal's body, potentially leading to their secretion into milk (Monteverde et al. 2022). According to Table 3, Pb concentration in 8% (2 samples) and 4% (1 sample) samples of spring and summer seasons, respectively, was higher than the maximum permissible limit. Almost 12% (3 samples) of spring samples and 4% (1 sample) of summer samples had Cd concentration higher than the maximum permissible limit. The results of the current study are lower than the results of Feizi et al. (2022) in Gorgan city, Iran, Yasotha et al. (2021) in India, and Koyuncu and Alwazeer (2019) in Turkey. The results showed that the mean concentrations of Pb and As were higher in milk samples collected during summer compared to spring, while Cd levels were higher in spring (*p* > 0.05). The detection of arsenic in milk may be attributed to water pollution, unsanitary production conditions, chemical contamination of equipment, or the application of pesticides and herbicides in farming (Beikzadeh et al. 2019). The higher contamination rate in spring milk samples could be related to the lack of fresh fodder to feed animals.

**TABLE 3:** Concentration of heavy metals (mg/L) in raw milk samples.

Heavy metals (mg/L)	Season	Min‐ Max	Mean ± SD
Pb	Spring	0.002–0.035	0.007 ± 0.009^a^
	Summer	0.002–0.09	0.014 ± 0.021^a^
Cd	Spring	0.002–0.058	0.011 ± 0.021^a^
	Summer	0.002–0.045	0.005 ± 0.011^a^
As	Spring	0.002–0.052	0.008 ± 0.013^a^
	Summer	0.002–0.02	0.011 ± 0.006^a^
Hg	Spring	0.002–0.008	0.002 ± 0.001^a^
	Summer	0.002–0.003	0.002 ± 0.0008^a^

*Note:* Different letters in two seasons show significant difference at *p* ≤ 0.05. LOD of As = 0.698 ppb, Cd = 0.665 ppb, Pb = 1.87 ppb, Hg = 0.541 ppb.

In the current study, the mean concentration of Pb, Cd, As, and Hg in spring and summer was 0.007 and 0.014 mg/L, 0.011 and 0.005 mg/L, 0.008 and 0.011 mg/L, and 0.002 and 0.002 mg/L, respectively. The mean concentration of Cd and Pb in milk samples of East Azerbaijan province (Iran) was 0.007 and 0.01 mg/kg, respectively (Safaei et al. 2021). The mean concentration of Pb and Cd in pasteurized milk gathered from Gorgan city of Iran was estimated at 0.02 and 0.023 μg/g, respectively (Feizi et al. 2022). The Pb, Cd, and As concentrations in milk samples of Tabriz (Iran) were reported as 6.066–10.83, 2.343–6.07, and 3.246–7.536 μg/L, respectively (Beikzadeh et al. 2019). Pb concentration in milk samples of Tehran province (Iran) ranged from 0.003 to 0.19 mg/mL (Sharifi et al. 2022). According to the results of Su et al., Pb, Cd, and As in raw milk samples gathered from China were ND–15.22, 0.02–0.39, and 0.1–1.49 μg/kg, respectively (Su et al. 2021). The mean concentration of Pb, Cd, and As in milk samples of Central Andean Areas was estimated at 0.062, 0.014, and 0.03 mg/kg, respectively (Castro‐Bedriñana et al. 2023). The variation in heavy metals content in different studies could be related to farm location, contamination of feed and water, traffic intensity, variation in production process, lactation stage of mammals, air contamination (especially lead), and distance to pollution regions (Su et al. 2021; Safaei et al. 2021).

## 4 Conclusions

The occurrence of aflatoxin M_1_ (AFM_1_), organochlorine pesticides (OCPs), and heavy metals in milk is influenced by multiple factors, including dairy cow physiology (lactation stage), environmental conditions (temperature and humidity), and farming practices (feed quality, pesticide use). Although the measured contaminant levels in this study were within the regulatory limits set by Codex Alimentarius and ISIRI, notable variations were observed due to seasonal fluctuations, geographical differences, and agricultural management. To minimize the contamination risks, different strategies such as proper feed storage (moisture control, mold prevention), use of mycotoxin‐binding adsorbents, and optimized post‐harvest handling should be implemented. Additionally, routine surveillance programs and strict compliance with international safety standards are essential to safeguard milk quality. Ensuring low contaminant levels not only protects consumer health but also strengthens public trust in dairy products.

## Author Contributions

**Farnoosh Ansari:** conceptualization (equal), data curation (equal), formal analysis (equal), investigation (equal), methodology (equal), software (equal), supervision (equal), writing – original draft (equal), writing – review and editing (equal). **Elaheh Askari:** conceptualization (equal), data curation (equal), formal analysis (equal), investigation (equal), project administration (equal), resources (equal), supervision (equal), validation (equal), writing – review and editing (equal). **Hamdollah Naderi Boroujeni:** data curation (equal), formal analysis (equal), investigation (equal), methodology (equal), project administration (equal), resources (equal), software (equal), validation (equal), writing – review and editing (equal). **Maliheh Jahanara:** conceptualization (equal), funding acquisition (equal), investigation (equal), methodology (equal), project administration (equal), resources (equal), software (equal), supervision (equal), validation (equal), writing – review and editing (equal). **Bita Forootani:** conceptualization (equal), funding acquisition (equal), investigation (equal), project administration (equal), software (equal), supervision (equal), validation (equal), writing – review and editing (equal). **Elham Khalili Sadrabad:** conceptualization (equal), formal analysis (equal), investigation (equal), methodology (equal), project administration (equal), supervision (equal), validation (equal), writing – original draft (equal), writing – review and editing (equal).

## Conflicts of Interest

The authors declare no conflicts of interest.

## Data Availability

Data available on request from the authors.
